# QED disinfection of Ebola and drinking water in the developing world

**DOI:** 10.1186/2047-2994-4-S1-I7

**Published:** 2015-06-16

**Authors:** T Prevenslik

**Affiliations:** 1 QED Radiations, Hong Kong, China

## Introduction

The UV disinfection protocol for Ebola and drinking water in the West [1] is not only too complex and expensive to be used in the developing world but requires sources of electricity usually not available.

## Objectives

To provide people in the developing world with a means to disinfect both the Ebola virus and drinking water themselves using UV-C radiation from hand-held nano-coated bowls powered only by body heat.

## Methods

QED induced EM radiation [2] from body heat in hand-held nano-coated bowls is proposed to disinfect the Ebola virus and drinking water. QED stands for quantum electrodynamics and EM for electromagnetic. By this theory, heat from the hand cannot increase the coating temperature because its heat capacity vanishes by quantum mechanics. Instead, body heat is conserved in the nano-coating by QED inducing the creation of EM radiation having wavelength λ depending on the coating thickness d and refractive index n, i.e., λ = 2nd. For example, a bowl comprising a thin-walled aluminum half-sphere (100 mm diameter x 50 mm high) that fits in the palm of one hand is provided on the inside surface with a 53 nm zinc-oxide coating having n = 2.4 to produce the UV-C. Humans produce body heat of about 6 mW/cm^2^. Since the UV-C intensity necessary to disinfect [3] the Ebola virus is 0.4 mJ/cm^2^, the protocol is to move an empty inverted hand-held bowl over the area to be disinfected in < 1 second scans. Water disinfection requiring 16-38 mJ/cm^2^ of UV-C [4] means filling the bowl with water and waiting 3 to 6 seconds before drinking.

## Results

Preliminary results expected for the ICPIC conference.

## Conclusion

QED induced UV-C radiation from hand-held nano-coated bowls allows people in the developing world to rely on themselves to disinfect the Ebola virus and drinking water. Costs of the bowls are minimal and may be distributed freely by West African governments to their people. Support and funding in the development and testing of UV-C disinfection of Ebola and drinking water by the Innovation Academy is requested.

## Disclosure of interest

None declared.
